# Required and desired: breakthroughs for future-proofing mineral and metal extraction

**DOI:** 10.1007/s13563-022-00328-0

**Published:** 2022-07-13

**Authors:** Elisabeth Clausen, Aarti Sörensen

**Affiliations:** Institute for Advanced Mining Technologies (AMT), RWTH Aachen University, Aachen, Germany

**Keywords:** Mining, Digitalisation, Metals

## Abstract

The global industrial mining sector is, like other sectors, undergoing an unprecedented transformation pushed by global sustainability and climate challenges. The need to increase productivity and efficiency of mineral extraction along with increasing pressure from a wide range of stakeholders to decarbonise the industry and make mining practices more sustainable, accountable, and socially acceptable are driving the adoption of automation and digitalisation technologies as well as the electrification of equipment and the implementation of more sustainable energy solutions for the industry. Automation and digitalisation are changing the way minerals and metals are extracted and provide important tools for designing and implementing the mine of the future: a digitally integrated, autonomous mine where no humans need to be put in harm’s way and in which the connected systems are able to reduce the ever-increasing complexity to such an extent that improved decision-making can be realised in real time. Mining as an industry still has a way to go to reach the potential of automation and digitalisation on the one hand, and alternative drive systems and sustainable power generation on the other. This paper will give an overview of empirically derived leading technologies underlying the current transformation and will place them in the context of the data-information-value-chain that can provide a more systematic approach to describe the various technologies and, in particular, their interrelationships. This can support a better understanding of assessing the overall technological maturity of an operation, especially with respect to their evolution from automation of equipment towards autonomous systems. There is no reason to doubt that, from a technology perspective, the digitally connected, autonomous, and carbon-free mine have the potential to become a reality. Breakthrough effects can be expected to come not from any single technology but rather from successfully developing, implementing, and integrating the full suite of (available) automation and digitalisation technologies across entire mining operations and moving towards digitally integrated, autonomous systems considering the process and its interrelations holistically (Clausen et al. 2020b). However, in order to get there, mining companies need to consider not only the technological aspects of this transformation. For successfully responding to the changing landscape of stakeholder expectations and future-proofing the industry requires, the authors argue that mining companies need to adopt a mind-set of the human-centred climate smart mine (Clausen and Sörensen 2021). In addition, mining companies need to reconsider their role in the economic, social, and environmental ecosystem they are embedded in so they can break through traditions that keep them from successfully positioning themselves as builders of social value.

## Introduction

The production of raw materials is indispensable for economic growth and welfare. Even more so, a broad range of metals and minerals is required for achieving the transition towards greener and carbon-free economies as well as for the development of future high-tech applications. With the projected growth in demand for metals and minerals in the coming decades, which cannot be met even if recycling rates substantially increase, the mining industry, for the foreseeable future, will remain the leading producer of the materials we need to build our low carbon sustainable future (IEA and International Energy Agency 2021).

The mining industry is not only providing the building blocks for a more sustainable future, it directly and indirectly contributes to achieving the Sustainable Development Goals (SDGs) (World Economic Forum 2016). Even though the mining sector is a significant emitter of greenhouse gases and often located in ecologically sensitive and less-developed areas including indigenous lands and territories, it does have the opportunity and potential to positively contribute to all 17 SDGs. This can be achieved, for example, through a commitment to provide inclusive employment opportunities, support local supply capacity, and strengthen local procurement, as well as investments in renewable energy and shared infrastructure or infrastructure linkages with agriculture and local communities, and increased financial transparency. It is important to note that positive contributions by mining companies can include improvements towards the SDGs and corresponding targets as well as mitigating or preventing negative impacts on the SDGs (World Economic Forum 2016). However, in order to actively contribute to achieving the SDGs, the mining industry “must ramp up community engagement, partnership and dialogue with other industry sectors, government, civil society and local communities” (World Economic Forum 2016).

It is precisely these rising expectations of a wide range of stakeholders that has moved the global mining industry to the spotlight and put increasing pressure on performing not only economically, but also socially and environmentally in a more sustainable way. Aside from well-known industry inherent challenges of increasing productivity and operational efficiency due to lowering ore grades, farther transportation routes and, consequently, increasing development and production costs, this value shift that has taken place in recent years means that sustainability is no longer a “nice-to-have” but has become imperative for long-term competitiveness and existence of mining companies (Clausen et al. 2020b; Ellis 2020; Durrant-Whyte et al. 2015; Mitchell 2021). Investors are increasingly judging companies on factors such as their environmental footprint, carbon footprint, greenhouse gas emissions, and energy consumption, as well as their safety record and benefits to employees (Burns 2020). Companies with higher ESG ratings outperformed not only during the peak of the COVID-19 pandemic but also in the longer term (PwC 2021). In addition, (end-) consumers increasingly demand products coming from traceable sources produced in a responsible manner that they offer fair and secure jobs to their employees, and that they protect the environment and support the communities in which they operate (Clausen et al. 2020b).

Notwithstanding the technological challenges of digital transformation, the real game changer in mining, therefore, can be considered to be the changing societal values and shifting stakeholder expectations, embedded in the larger transformation towards low-carbon economies to mitigate climate change. It means that mining companies have to rethink and redefine their role within society. The mining industry needs to align with the values of today’s generation, which include transparency, responsible technological innovation, sustainability, and shared prosperity. This means that mining companies need to rethink how they can and will create sustainable value for all stakeholders (Jamasmie 2020).

Technological innovation and the transformation towards increasing automation, digitalisation, and electrification present important enabler, not only for improving economic performance and ensure mining operations to remain profitable but also for meeting sustainability targets and stakeholder expectations. However, to date, the discussion on technological innovation in mining has strongly focused on improving economic parameters and safety (Barnewold and Lottermoser 2020; Sánchez and Hartlieb 2020). The concept of Mining 4.0, or the Smart Mine, has been established as a technological transformation driven by digitalisation and automation, and to some extent electrification, in the mining sector.

Against the backdrop of the wider societal challenges around climate change and sustainability, however, the concept of Mining 4.0 should be expanded to more explicitly include social and environmental dimensions. While technology is an important enabler for change, it should and cannot be considered independently from its purpose and its impact on miner’s triple bottom line to ensure continued trust of stakeholders and thus ensure that mining companies can thrive alongside the societies and communities they operate in. Therefore, mining companies and researchers could benefit from an expanded notion of Mining 4.0 to make their efforts more explicit to make mineral extraction not only more efficient and safe, but also environmentally friendly, and more socially acceptable.

Furthermore, the concept of Mining 4.0 encompasses an understanding of the interrelationships between technologies and thus aims for a more integrated view of technological transformation. Recent publications on innovation and digitalisation tend to give anecdotal examples for the implementation of specific digital or automation solutions without providing deeper insight on how these technologies build on each other and relate to one another. For example, when considering battery-electric equipment, rather than just replacing diesel equipment with electric vehicles, the electrification of mines and elimination of greenhouse gas emissions needs to include a redesign of processes and also include the dimension of energy supply to the mine site.

Therefore, this paper aims to introduce an expanded notion of Mining 4.0 to include a range of non-technological dimensions and to frame this expanded concept as the “Human Centered Climate Smart Mine”.

In the following paragraphs, first, technological dimensions and challenges will be outlined, leading technologies of Mining 4.0 introduced, and a more integrated understanding of the technologies commonly subsumed under the term “Smart Mining” or, respectively, Mining 4.0, will be presented using the data-information-value chain. Secondly, various non-technological dimensions that should be considered in conjunction with the technological transformation are introduced and discussed. The conclusion provides a summarising argument for the “Human Centered Climate Smart Mine”.

## Technological dimensions of change

When talking about technological dimensions as enabler for the transformation of mining operations, automation, digitalisation, and electrification are nowadays considered as the main drivers and trends. Efforts to automate and electrify mining processes have existed not least since the third industrial revolution, even though electrification and the use of alternative drives have been stepped up in recent years, particularly with regard to the increased use of battery-powered electric vehicles, against the background of reducing mining-related CO_2_- emissions and contributing to climate protection. Developments towards digitalisation and digital transformation are currently driven as part of the fourth industrial revolution initiated around ten years ago and characterised by intertwining industrial manufacturing with advanced information and communication technologies. For the mining industry, this concept for transforming the industry towards Industry 4.0 in terms of “Mining 4.0” or “Smart Mining” can be understood “as the intelligent connection and integration of mining machines (physical components) (with) (…) information and communication technologies (cyber- systems) to form so-called Cyber-Physical Systems, (where) (…) the exchange and transmission of data and information take place via a platform, the Industrial Internet of Things (IIoT)” (Clausen et al. 2020a). Essential components comprise sensors and actuators as physical objects, process modeling, (real-time) simulation, optimisation, and computer networks as cyber elements embedded in a system characterised by context management and object relationships along the value chain and integrated via computation, communication, and control technologies. Due to the nature of physical processes, dynamics and timing are important requirements, which leads to challenges in terms of standardisation of processes and interfaces and the integration of the individual components. Digitalisation in mining, at its core, encompasses the vision of a digitally connected, autonomous mine in which connected systems are able to reduce ever-increasing complexity so that real-time decision-making can be realised. While various technologies have been developed over the past decade in terms of increased computing power and capacity, storage capacity, and improved sensor and communication technologies, mining is still in the early stages of realising this vision.

The mining industry itself is generally considered a late adopter of digital technology and lagging behind other industries, such as oil and gas, automotive, or chemicals (Ganeriwalla et al. 2021). Yet, several mining houses have made substantial investments in automation and digital technologies over the past decade. Overall productivity has increased since 2012 after a decade long decline and it seems that these investments have created an impact. However, according to McKinsey, only some companies have seen the desired results, while others did not (McKinsey & Company 2018). Consequently, industry-wide adoption and diffusion of digital technologies across the mining sector has remained comparatively low to date (Barnewold and Lottermoser 2020).

The reasons for low diffusion of available technologies are, of course, multidimensional. On the one hand, implementing the mine of the future presents challenges that are specific to the mining industry. Besides the known reasons for general reluctance towards risk and innovation by a generally volatile and cost-intensive industry, this includes the fact that technologies developed in other industries cannot easily be transferred to the mining conditions. Due to the harsh and complex conditions in mines, many of the technologies must be developed or adapted specifically for the mining environment. In addition, solutions often have to be customised as use cases vary greatly among operations. These factors need to be considered when evaluating and comparing the digital transformation in mining to other sectors.

On the other hand, several reasons for unsuccessful digitalisation initiatives have been identified, among them the lack of an overall vision or roadmap of transformation before choosing the technologies to be implemented as well as a gap between strategy and execution and not making required adjustments in company culture and management systems (McKinsey & Company 2018; Ganeriwalla et al. 2021; Monitor Deloitte 2016). While this partially explains why some companies have been more successful than others, these (non-technical) issues further exacerbate the low diffusion, hesitant adoption, and low impact of new technologies across the sector.

Focusing on the challenge of selecting the right and most suitable technologies for a specific mine site, ideally based on a vision and roadmap for transformation, it is an important success factor to understand, which technology is useful and applicable at what stage of the digital transformation or automation journey and a more systematic approach to technology adoption can serve as a backdrop for designing roadmaps for digitalisation and automation projects.

Even within the scientific community, digital technologies and their adoption in mining are mostly discussed anecdotally rather than systematically (Sánchez and Hartlieb 2020) or the technology diffusion empirically assessed without discussing the interrelationships between technologies and how they may build upon each other (Barnewold and Lottermoser 2020).

### Leading technologies of digital transformation

A recent comprehensive research study on the impacts of digitalisation technologies on sustainability aspects in the global mining industry identified 15 core technologies to be leading the current wave of digital transformation (Clausen et al. 2020c).

According to the study, which was based on a literature review and semi-structured interviews of global experts, the technologies mostly mentioned and discussed as currently having an impact on changing the way mineral and metal extraction is done are as follows: automation, IIoT, remote operation centres, connected worker, robotics, drone technology, electrification, 3-D printing, integrated software platforms, advanced analytics, simulation and visualisation, cloud computing, big data management, cybersecurity, and E-learning. These results are largely consistent with other studies in this area (Barnewold and Lottermoser 2020).

**Fig. 1:**
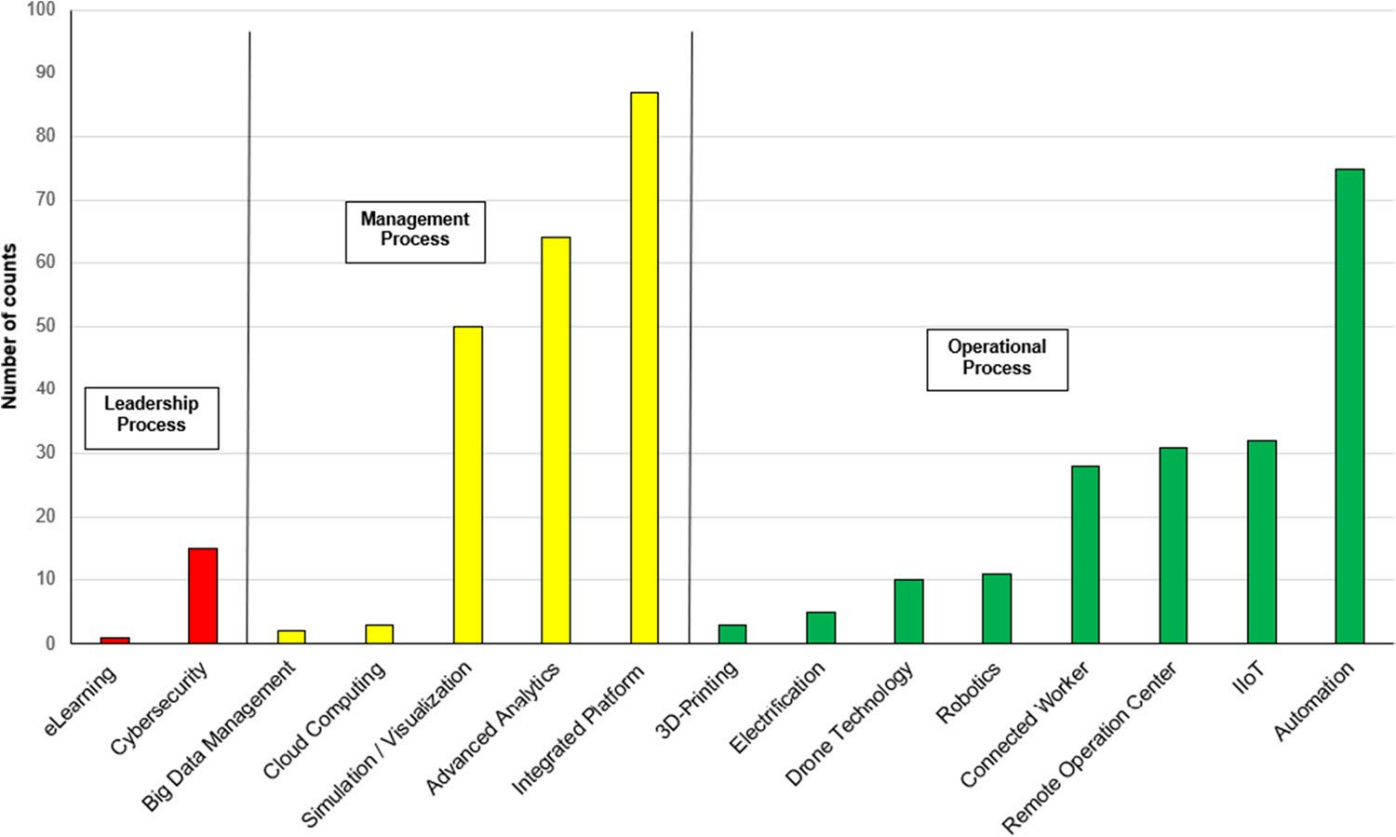
Digitalisation technologies on organisational levels based on literature review (Clausen et al. 2020b)

Figure 1 shows the percentage of the technologies being mentioned in the sources that were analysed for the study. These technologies are impacting different organisational levels within the organisation. The study differentiated between operational (green), management (yellow), and leadership (red) process levels. The leadership processes included “corporate and governance activities involving multiple operations of a mining company as well as the C-Suite level”, the management level “organisational activities and personnel including planning, coordination, control and adjustments of the production process and allocation of resources including equipment utilisation and work plans”, and the operational process level core mining processes (Clausen et al 2020a). The core mining processes itself have been further differentiated into development, extraction, ventilation, rock and roof support, haulage and transportation, maintenance, backfilling, and water and waste management.

Out of these 15 technologies, as can be seen in the graphs above, the most mentioned ones the study identified are:

- Automation
- Industrial Internet of Things (IIoT)
- Remote operation centres
- Connected worker
- Integrated platforms
- Simulation and visualisation
- Data analytics

Aside from identifying the most prominent technologies, the study showed that automation, IIoT, remote operation centres (ROCs), and connected worker are associated with impacts on the operational process levels. These technologies are aimed at improving safety of the workers by removing people from high-risk areas and monitoring their health parameters and location. Furthermore, automation, IIoT, and ROCs are aimed at improving productivity and reducing operational costs at the operational level.

With respect to the management processes, the study showed a strong impact by digitalisation technologies, namely integrated software platforms, visualisation and simulation technologies, and data analytics. The data generated at the operational level is being brought together at the management level in order to gain insight into and adjust processes, production rates, equipment utilisation, etc. within short timeframes or close to real time.

For leadership processes, cybersecurity was most mentioned during the literature review and is expected to be a major concern for company leadership suites in the coming years.

### Interdependencies of technologies

**Fig. 2:**
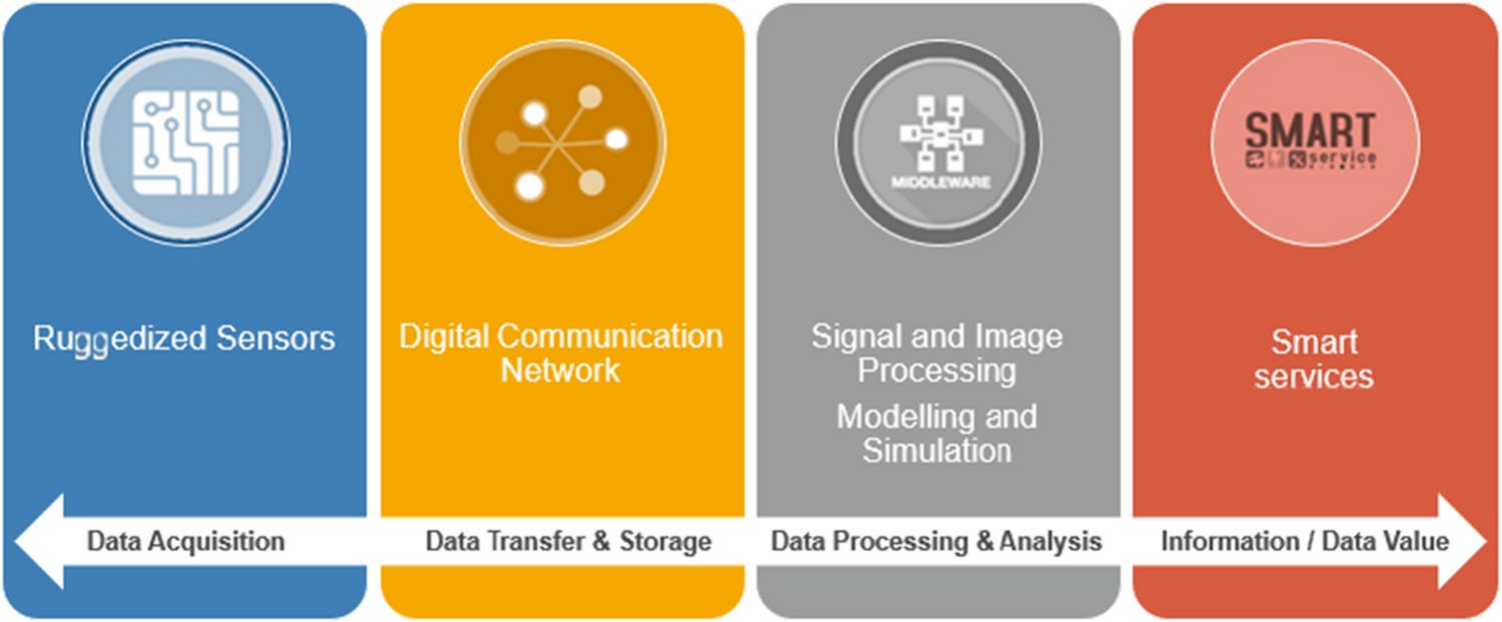
Data-information-value chain adapted from (Clausen et al. 2020)

For the definition and description of interdependencies of technologies, the approach of the data-information-value chain (Fig. 2) is used, where the transformation from data into relevant and useful information forms the basis for value creation for several applications. At the beginning, there is a data source for the acquisition of data, which can comprise data from physical objects (sensors) and/or derived from modeling and simulation. These data sets are transmitted via communication technologies and stored for making them accessible for subsequent processing and analysis. This may include signal and image processing or modeling and simulation to analyse and evaluate the data for transforming it into information, which can then be used specifically and purposefully for various applications as “Smart Services”. Related to cyber-physical-systems in mining these smart services could range from information for decision support to autonomous actions (Clausen et al. 2020a).

**Fig. 3:**
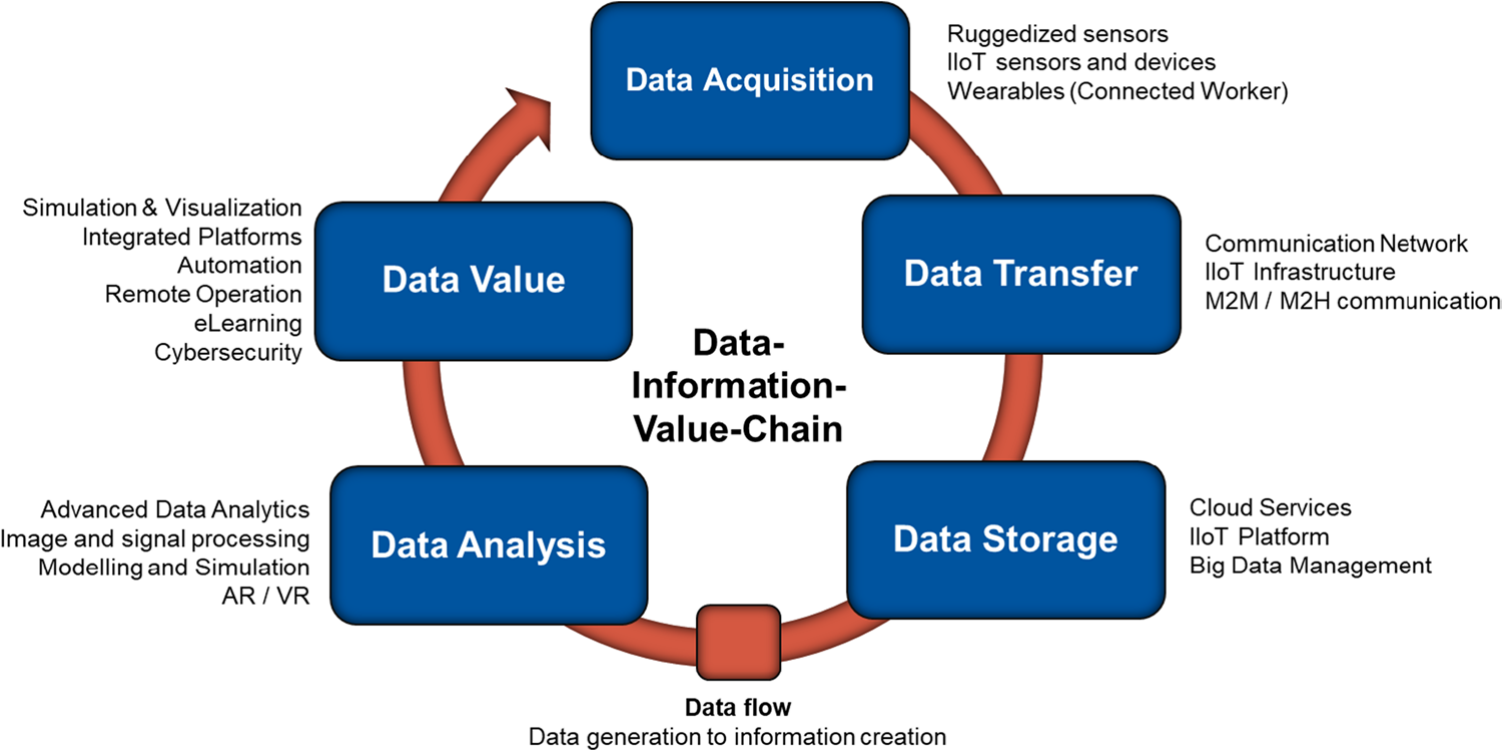
Data-information-value-chain and related technologies (Clausen et al. 2020)

This data-information-value chain can provide a useful backdrop for a more systematic approach to analysing digital technologies and their interrelationships. This model also allows to take the technologies broadly discussed in literature and press and put them in a systematic and structured context. In Fig. 3, the respective technologies reviewed and that were identified in the study are placed along this data-information-value chain model.

What Fig. 3 indicates is that many of the technologies that are considered to have a strong impact on the industry have a number of prerequisites that need to be met in order for them to unfold their potential.

Taking automation as an example, which is considered the most impactful technology in increasing productivity, improving safety and lowering operational costs to date. Yet, there are a substantial number of prerequisites that need to be met in advance. At the stage data acquisition, ruggedised or virtual sensors need to be in place that collect the primary data. The type and quality of the data acquired at this stage are essential for the value that can be created from the data as information further along the value chain. Secondly, the sensor data needs to be transferred via a robust and reliable communication network and, in a third step, stored in a platform or cloud service centre making the data available and accessible for various applications. In a fourth step, the data is analysed and utilised to generate useful information that then can be connected to the process or machine in a fifth and last step, which then lead to the actuation.

This means that ruggedised sensors form the basis for any kind of automation, but also for remote operation, and digitalisation technologies such as advanced analytics and simulation and visualisation, like digital twins. They generate the data that is the basis for increased information about the operation and all subsequent automation and optimisation processes. However, it is precisely the type and quality of data, hence the performance of sensors, and the decisions on what sensors to apply and where they should be applied for the best results, that is still posing challenges for tapping the potential of automation and digitalisation.

Consequently, further down the data-information-value chain, another challenge that is hampering the potential of digitalisation is the creation of value from the available data that can be visualised and support decision-making processes effectively. To date, only little of the data that is available can actually be utilised meaningfully in the operation. According to estimates by McKinsey, less than 1% of the available data is utilised for actual decision-making at the execution stage (Durrant-Whyte 2015). Precisely because the quality of the data produced, analysing it in real time or short intervals in order to derive conclusions than can help improve decision-making and optimise processes still remains a great challenge for deriving benefits for the operation as well.

Another factor that is limiting the potential of advancing automation towards autonomous systems is the challenge of creating compatible interfaces between machines and systems in order for them to communicate and be integrated into a single platform or interface. However, machines and systems often lack interoperability as suppliers often use proprietary technology solutions not compatible with other systems in the operation (Aziz et al. 2020).

Developing and applying suitable sensors is still a major challenge for mining companies, especially in underground environments, followed by challenges and impeding costs of implementing an all-encompassing communication network and IIoT infrastructure across the operation. These challenges affect the effectiveness of automation and digitalisation efforts further down the data-information-value-chain and partially explain why operations have not been able to tap the full potential of the available technologies. Furthermore, this selection of a suitable set of technologies is still only possible to a limited extent, as the related impact is often not available or not even known due to a lack of a wide range of references.

### Current levels of implementation

While the type of commodity does not have any impact on the level of implementation of digital technologies, surface operations tend to be more digitally advanced than underground operations, due to particular challenges of bringing digital technologies underground. Digitalisation initiatives itself tend to be mostly driven by multi-asset and often multinational corporations with large-scale operations across the globe, even though some mid-tier corporations set benchmarks through agility and cost-effective innovations (Clausen et al 2020b).

The reality in most operations to date, though, is that technology implementation looks more like, metaphorically speaking, a patchwork quilt, and the autonomous mine is still a vision of the future. This is because the installation of comprehensive IIoT infrastructure encompassing the entire operation is costly and, depending on the location of the mine site, difficult to implement. However, this means that the companies cannot take full advantage of digital connectivity if only some parts of the operation or certain pieces of equipment are connected. It is to be expected, though, that further advances in comprehensive communication infrastructures will be realised in the coming years and thus enable mining companies to take full advantage of digital connectivity.

Another reason is that in most cases, mining companies implement selected technologies in a discrete manner, meaning disparate solutions that still exist more or less isolated from each other and are not necessarily implemented across the entire operation not yet part of an integrated system. In addition, not all available technologies usually all get implemented in one operation. For example, one operation focuses on implementing automated drilling, while others focus on automated haulage or battery-electric trucks. Some operations have implemented extensive communication infrastructure and have focused on wireless connectivity for improved oversight of the operation in real-time.

Consequently, the potential of existing technologies, especially that of integrated platforms and advanced automation towards autonomous systems, has not yet been realised widely across the industry (Clausen et al. 2020b). This is further confirmed by the analysis of Barnewold and Lottermoser that have empirically shown that the diffusion of most technologies is still well below 5% (Barnewold and Lottermoser 2020). Hence, while productivity improvements have been achieved over the past decade, more significant breakthroughs in productivity and efficiency as well as in safety and environmental performance cannot yet be expected at this stage.

Considering that, to date, less than 1% of the available data is utilised for actual decision-making at the execution stage (Durrant-Whyte 2015), gaining real value from the available data to support management processes and successfully applying advanced analytics and utilising the data to manage and control the operation through integrated software platforms is still posing a major challenge for most mining companies. However, it can be expected that once the data provides valuable and actionable insights on a consistent basis, advanced analytics and integrated platforms will transform the industry and its performance. Once usable data becomes available across the operation, integrated optimisation approaches that consider the entire value chain are conceivable and bear the potential to be one of the prospective key trends with great potential for productivity and efficiency gains (Clausen et al. 2020b).

With respect to advancements towards autonomous systems, some of the core technologies, such as ruggedised sensors for harsh environments and interoperability interfaces, technology for machine-to-machine communication, and autonomous navigation in GPS-deprived underground environments, autonomous and robotised inspection technologies, and online material characterisation still need to be developed and brought to market. It is important to emphasise that technologies that will enable advanced automation and autonomous systems are not yet fully developed, especially for underground operations and are still at the development, testing, and demonstration stage.

**Fig. 4:**
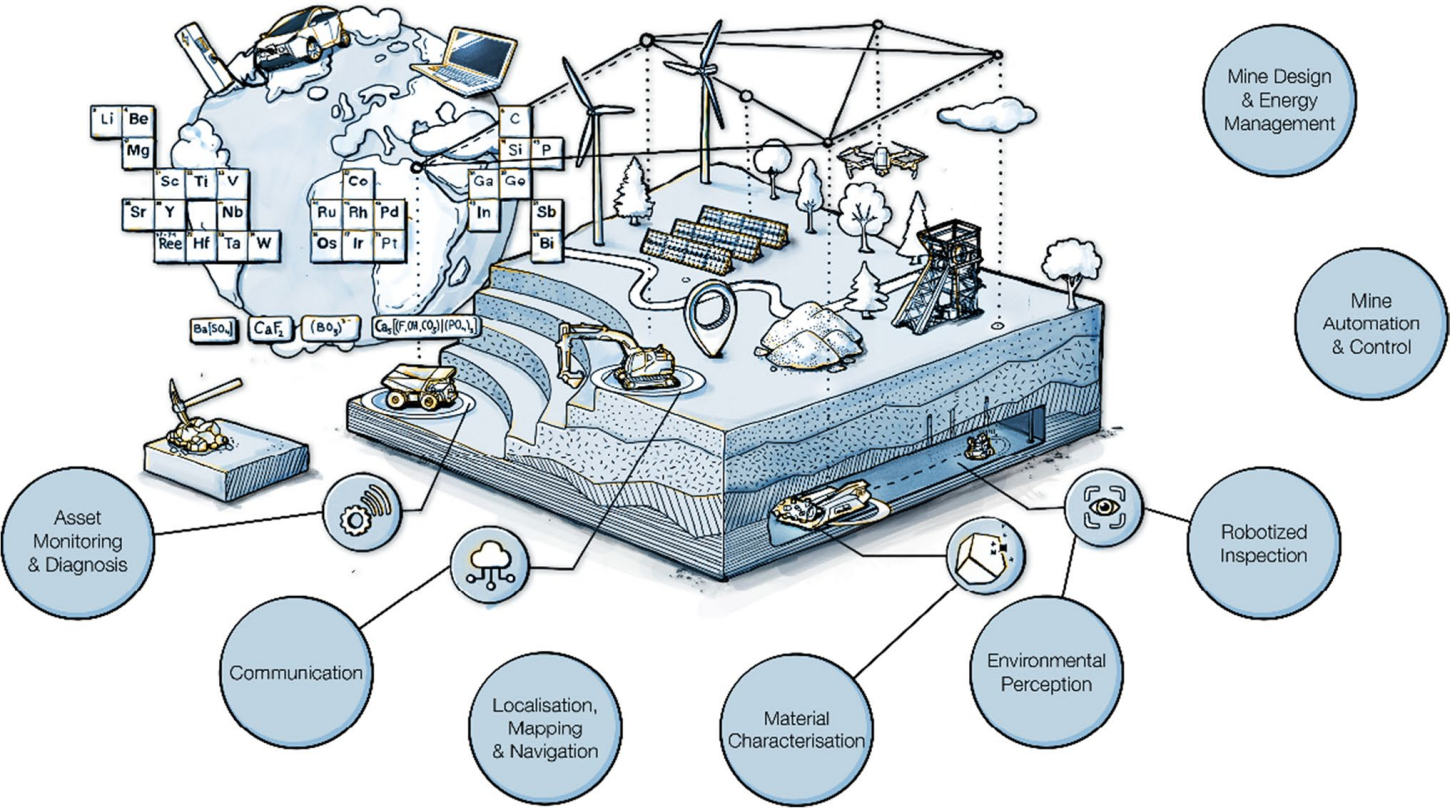
Technologies for the autonomous mine of the future

Some of the key technological components for autonomous mining systems are shown in Fig. 4.

These components need to be further developed and matured individually and then integrated into a comprehensive system in order to move towards the vision of the autonomous and green mine of the future.

Another challenge that needs to be resolved in order to progress towards autonomous systems and that is slowing down the implementation of digitalisation and autonomous systems is a lack of interoperability between machines and the lack of compatibility between different systems. What is required is the creation of an open interface standard that defines the mechanisms of cooperation in the industrial environment, especially between machines. To this end, VDMA Mining and the OPC Foundation, together with the Institute for Advanced Mining Technologies and supported by numerous VDMA member companies, have taken on the challenge to develop industry-specific OPC UA Companion Specifications for mining. The initiative is also supported by the Global Mining Guidelines Group (GMG), a not-for-profit membership organisation that provides a platform for collaboration and relationship building between stakeholders from within and across the mining industry. GMG thus “promotes the sharing of knowledge, expertise, and experience to develop operator-driven guidance, resources, and common practices that can be operationalized in response to some of the industry’s most pressing demands across the globe” (GMG 2022). As such, the GMG is a strong force in developing best practices and guidelines for the industry that can accelerate the adoption of new and innovative technologies and that are instrumental in moving ahead towards implementing autonomous systems.

Thinking developments towards autonomous systems further, it is possible to conceptualise intelligent self-organising robot swarms that will explore and even extract ore or break and leach it underground with very little impact on the environment, drones that perform in situ scanning, or deep-sea robots that mine underwater (Tauber et al. 2018). Furthermore, genetically manipulated bacteria or nanobots that mine at the molecular level are conceivable and water-neutral processing technologies are in development that may dramatically decrease the environmental footprint of mines (Tauber et al. 2018). Additionally, space mining, specifically in terms of in situ resource utilisation (ISRU), gained increasing interest in recent years (ISECG 2021).

### Case study: NEXGEN SIMS

One example of where some of these advanced technologies are currently being developed, tested, and demonstrated, while re-designing processes and thinking about technology in an integrated way, is the European H2020 NEXGEN SIMS project.

**Fig. 5:**
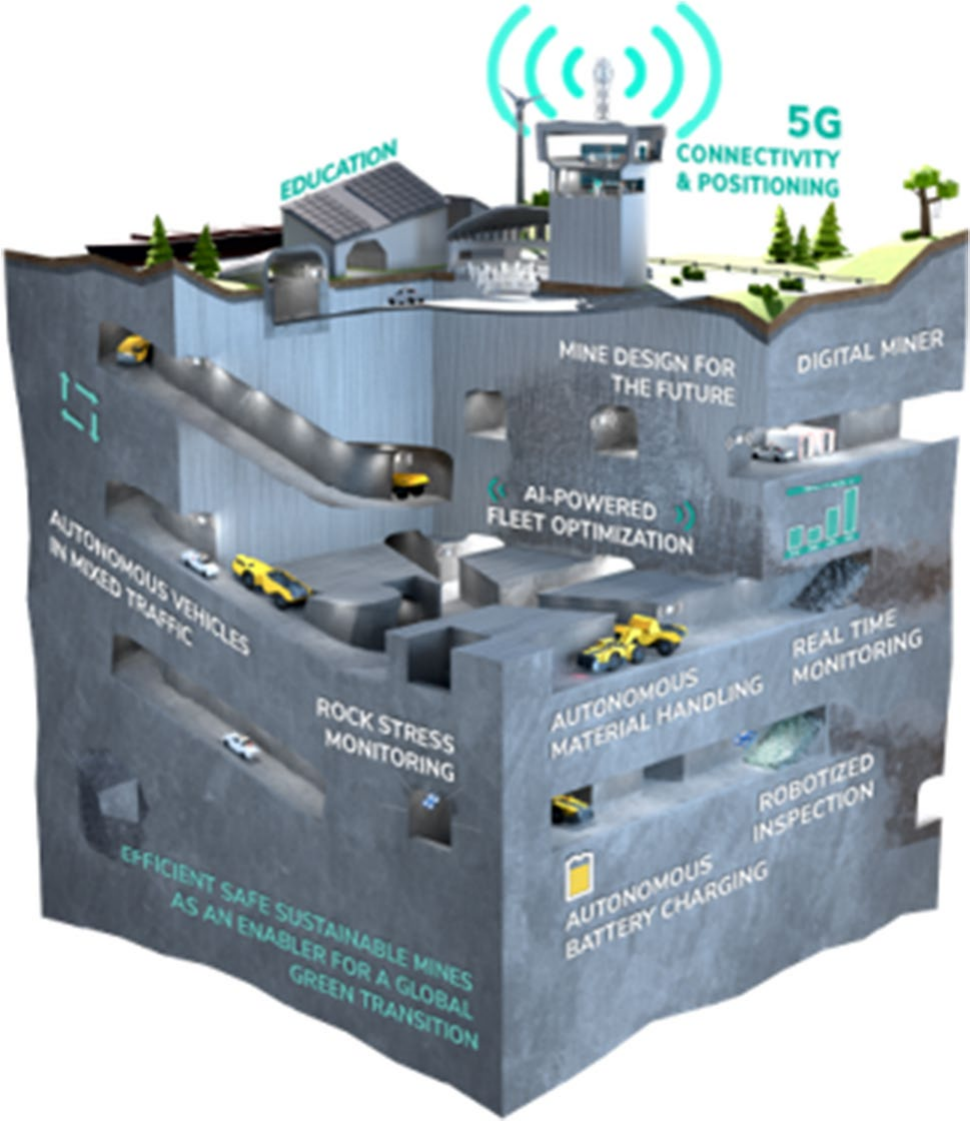
NEXGEN SIMS project overview: components of sustainable intelligent mining systems of the future (Nexgen SIMS 2022)

NEXGEN SIMS is a European Union-funded flagship project to support the development and demonstration of new technologies, methods, and processes that will enable more sustainable and efficient carbon neutral mining operations. An international consortium of mining companies, equipment and system manufacturers, and universities has started the three-year project in 2021. In order to advance autonomous carbon neutral mining processes, the use of battery-electric mining equipment, 5G for optimal connectivity and positioning, autonomous material handling, AI-powered traffic and fleet control, and collaboration among machines is being developed, tested, and demonstrated at selected mine sites by various project partners over the course of the project. The project also focuses on the mine worker of the future and safety aspects, for example by developing autonomous mine inspection technology. While the technologies are developed within various work packages by different constellations of partners, they are all guided by a unified vision of the sustainable intelligent mine as an integrated system, as depicted in Fig. 5 (Nexgen SIMS 2022).

The project is coordinated by mining equipment and service supplier Epiroc and the other project partners consist of mining companies Boliden, Agnico Eagle Finland, KGHM Polska, K + S, and OZ Minerals; services and system suppliers are Ericsson, Mobilaris MCE, AFRY, and KGHM Cuprum; business developer is LTU Business and universities involved are Luleå University of Technology and the Institute for Advanced Mining Technologies of RWTH Aachen University—all based in Europe, except for OZ Minerals which is based in Australia.

NEXGEN SIMS builds on the successful H2020 EU-funded SIMS project, which ran between 2017 and 2020 and played an important role in advancing sustainable mining operations, partly through the developed and deployment of a fleet of battery-electric machines developed by Epiroc over the course of the project (Paul Moore 2021).

The NEXGEN SIMS project provides insight not only into the complexity of technology development and integration of various technological solutions into an operation in an integrated, systematic, and connected way, but also into the importance of socioeconomic and environmental aspects that need to be put at the centre of why these technologies are developed in the first place.

The decarbonisation imperative is changing the parameters for mining companies and alternative drive solutions are of central concern, also to the NEXGEN project partners. Decarbonisation goes well beyond replacing diesel with battery-electric equipment; it means redesigning processes and rethinking energy generation and consumption at an operation-wide level, as done in the project as well.

The socioeconomic aspects are reflected not only in extensive measures to include and interact with the public to reshape the perception of mining but also in the concept of the “digital miner” that was developed as part of the preceding SIMS project and is being further elaborated in the NEXGEN SIMS project. The question of what the future workplace will look like and how digitalisation will impact the workplace from a worker’s perspective is central to the concept of the digital miner (Lööw and Johansson 2020; Halim et al. 2021; Lööw et al. 2019).

### Concluding thoughts on technologies – breakthroughs expected?

The mining industry is challenged with realising autonomous resource extraction that is safe, efficient, environmentally friendly, and socially acceptable while remaining economically profitable. This has become more of an imperative rather than a “nice to have” (Ellis 2020). While automation, remote operations, integrated IIoT platforms, simulation and visualisation technologies, and data analytics are already implemented at some mine sites and have made an impact, in most cases, only some of these technologies get implemented in one operation and, in most cases, not encompassing the entire operation (Clausen et al. 2020b).

In addition, the progression from automating pieces of equipment to fully autonomous systems, in particular, imply complex technological requirements, some of which are still in the development and/or demonstration phase. Examples include the development of robust sensor technology suitable for mining, the use of modern data processing and visualisation methods, the control and regulation of (automated or autonomous) machines and systems, including via man–machine or machine-machine interfaces, the development and implementation of suitable communications technology and standards, and a carbon neutral mine design.

These technologies need to be matured first, and then to be connected and integrated into the mining system. Using the analogy of a puzzle, where the pieces of the puzzle need to be built first, before they can put together. Once the “puzzle” will be further elaborated, there will be a sound technological backbone for the autonomous and green mine of the future. Against this backdrop, it will be possible to get a sound impression of the sustainability performance and capabilities of digitalisation at a specific mine site and to then consider the applicable technologies in a more systematic and integrated manner. If the right technologies are then combined purposefully and correctly, large benefits and even breakthroughs can be expected.

Technologies can only lead to “breakthrough” effects if they are targeted, which means value- and purpose-driven, and if they are aligned specifically to the respective process at a specific mining operation. This also means that the respective combination of technologies must be case-specific, targeted, and meaningful. It also means that various technologies may have to be developed or customised specifically for a particular application or operation as it will remain difficult to transfer technology from one mine to another without adapting it to the specific conditions of that particular site.

## Non-technological dimensions of change

The previous section discussed some of the leading technologies that are providing important pieces to the technological backbone of implementing the autonomous green mine of the future. These technologies are already having an impact on the industry at the moment and will likely create an even greater impact as they get implemented more comprehensively and systematically across operations.

The model of the data-information-value chain was presented to help map current technologies in a more systematic way. The discussion also showed that there is likely no single technology that will, on its own, create a breakthrough change. Rather, the breakthrough shift in the industry can be expected to occur once the leading technologies in the areas of digitalisation, automation, and electrification along with the necessary infrastructure get implemented comprehensively across entire operations. Furthermore, the previous section provided a roadmap for moving from automated machinery to autonomous systems as the next step towards the mine of the future that will lead to even more substantial breakthroughs with respect to achieving the vision of the autonomous green mine of the future.

Hence, from a technological perspective, there is little doubt that the digitally connected, autonomous, and electric mine will become a reality in industrial mining operations in the not too distant future.

However, there is another side to these technological dimensions of transformation: people. On the one hand, this includes people in the sense of stakeholder interests that constitute the socioeconomic context for miners to operate in, including governments that regulate the industry and request sharp declines in carbon emissions, for example, as well as investors that rate companies based on their ESG performance and benefits to employees, wider society, and NGOs requesting an increase of decarbonisation efforts, traceability of mineral production and responsible sourcing, and communities that are located in proximity of mining operations, as well as suppliers and partners within the company ecosystem.

On the other hand, this includes people who manage and work in a mining operation, as well as new talent moving into the industry, issues of management and company culture, which is created and shaped by the people managing and working in a specific company, and the ways innovation and transformation are driven and implemented within each company and new business models may be developed within or outside of the core industry.

**Fig. 6:**
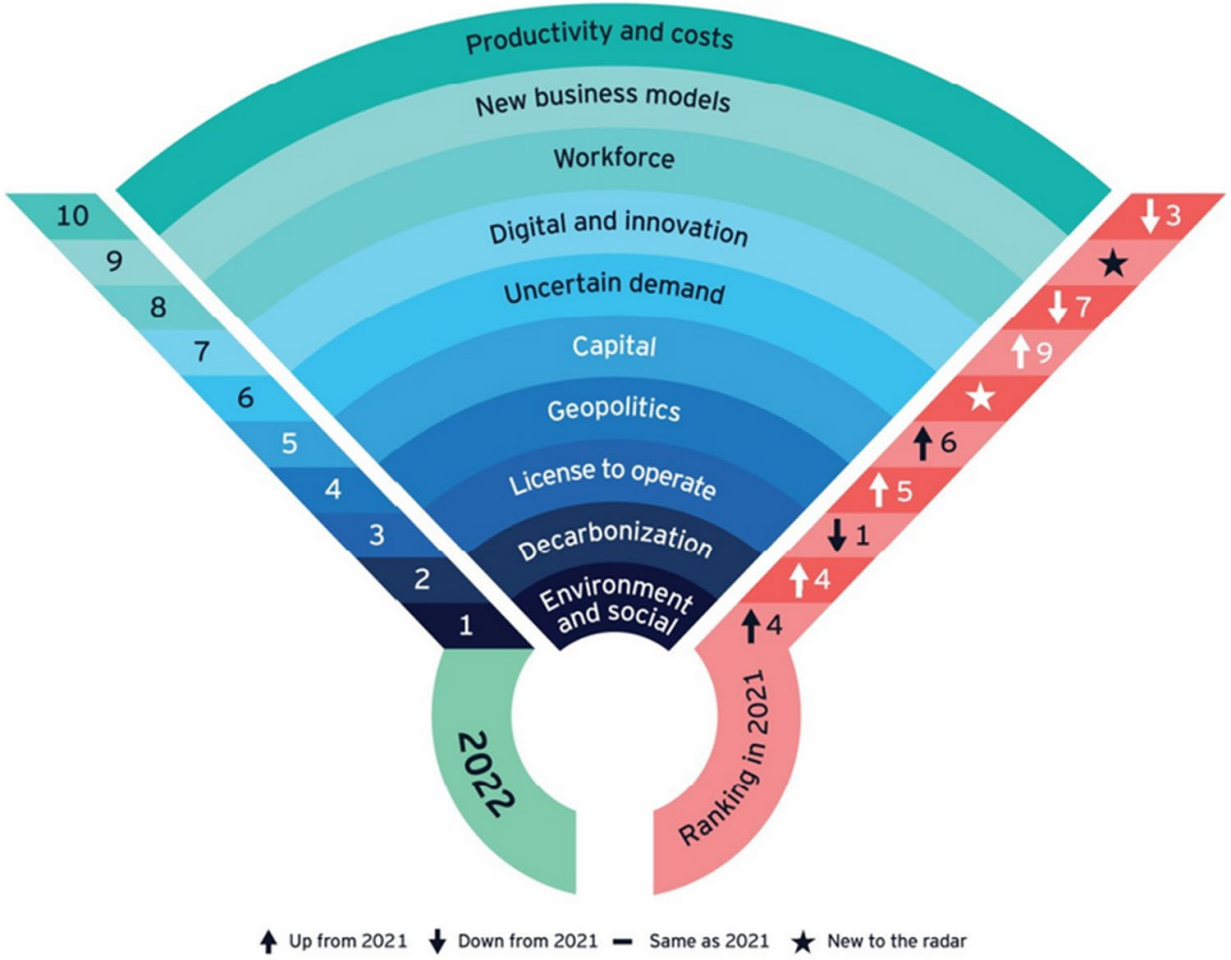
EY top 10 business risks 2022 (Mitchell 2021)

The fact that all of these aspects are no longer a “nice to have” but are becoming centre-stage in managing the future of the industry is becoming very clear when taking a look at the top 10 business risks 2022 by EY as depicted in Fig. 6 (Mitchell 2021).

Environmental and social issues, decarbonisation, and the social license to operate constitute the top 3 business risks today, whereas productivity and costs have moved to 10th place and digital and innovation to 6th place. The climate crisis and rising stakeholder expectations have become significant forces of change. According to EY, it is the first time in the history of the ranking that environmental and social issues, which include local community impact, water management, green production, diversity, and biodiversity as subcategories, have become the number one risk, whereas decarbonisation is considered “a major disrupter” that is now “dominating discussions, presenting both risks and opportunities” (Mitchell 2021).

While non-technological issues, such as social license to operate, decarbonisation, and sustainability, are being addressed extensively in mining media outlets as well as in academic papers,^1^ we would like to outline core issues in the specific context of technology development and innovation. We will focus on six areas that present important requirements to make the technological breakthroughs outlined above possible in order for the industry to advance towards autonomous, zero carbon mining.

Consequently, the following paragraphs will outline six areas that we consider relevant in the context of technological advancements in mining.

### Education for innovation

In order to foster technology development and innovation within the mining industry, new generations of engineers and talents also from other disciplines have to be attracted to the mining sector. This provides a challenge in itself given the widespread negative public image of mining companies and the idea of mining still being a dirty, dusty, and dangerous industry to work in. However, it is of great importance for the future success of the industry that a young and diverse workforce can be attracted into the mining sector in order to support the technological but also cultural transformation and reshape the face of the mining industry.

At the same time, these new generations of miners need to be equipped with the right skillset to drive the transformation and in order to provide leadership and direction. This is no small task since mining companies must continually evolve their operations with next-generation technologies to stay competitive and thrive, meaning that the increasing technological complexity of mining operations result in changing requirements for mining engineering education. This makes it necessary to adapt teaching and learning activities (Tauber et al. 2018; Valverde 2020).

Tomorrow’s mining engineers must be able to adapt to changing requirements of job profiles. This requires a strong foundation of disciplinary skills that enables graduates to respond to challenges in a flexible way. At the same time, in order to meet the challenges of a changing work environment, graduates need to develop problem-solving skills, a range of soft skills, and an entrepreneurial mindset that is conducive for innovation and enables young professionals to play a more active part in driving the industry transformation (Valverde 2020).

Entrepreneurship education is traditionally part of business and management education aiming at fostering business skillsets and business activity. However, a wider scope and more comprehensive definition of entrepreneurship education has evolved over the past decade. This wider concept of entrepreneurship education makes aspects of personal development, creativity, self-reliance, initiative taking, and action orientation, as well as in particular the aspect of creating value the main objective. With this focus on mindset instead of just skillset, it aims at enabling students to become more creative, opportunity oriented, proactive, and innovative and to manage and participate in value-creating processes (Bell and Bell 2020; Aldianto et al. 2018; Lackéus 2015; Sörensen et al. 2022).

This kind of entrepreneurial mindset can be realised by fostering problem-based and innovation-oriented teaching and learning through, for example, challenges, which can be integrated in already existing courses and expose the students at an early career stage with real industry cases. Additionally, it is important to provide suitable learning spaces and an environment conducive for innovation. In addition, partnerships between industry and universities play an important part in enabling actual value-creation processes for students that create actual benefits. Furthermore, these partnerships can advance innovation by applying academic research and development to actual industry problems as part of joint and applied projects (Siegel 2004; Valentin 2000).

### Partnerships and collaboration for innovation

After many of the large mining companies have substantially lowered their in-house R&D efforts and only “very few of the largest mining companies maintain in-house research groups but quite small if compared to the past”, which are usually then working on specialised technological solutions to specific issues, the sector now “finds itself at an impasse where innovations are mostly of an incremental nature …[and] the potential to drive breakthrough innovations [..] requires a more systemic type of collaboration within the framework of a well-functioning business” (Calzada Olvera 2022).

Consequently, partnerships and collaboration for innovation have taken on a new dynamic in some places as mining companies have started to look outward to supplement their internal innovation efforts and drive the industry faster towards a more sustainable future. Driven by the many challenges the mining industry is facing and which are too large and too urgent to be solved by the efforts of individual companies, mining companies have started to shift towards more collaborative R&D efforts, often through joining forces with OEM’s and tech suppliers in longer-term alliances.

In addition, there have been initiatives across the sector from mining companies, i.e. the “FutureSmart Mining” (Anglo American 2022) initiative by Anglo American, from governments, i.e. the “MICA” mining innovation acceleration network in Canada (MICA 2022), and multi-stakeholder initiatives, i.e. “Expande” (Expande 2022) mining innovation accelerator in Chile or the “ReThink Mining” (ReThink Mining 2022) in Canada, which are going even further and are aiming at building multi-stakeholder innovation ecosystems. These ecosystems for open-source collaboration involve mining companies and suppliers, as well as research institutions, universities, tech start-ups, and industry organisations, to accelerate new technology development and adoption in the industry (Leonida and Soerensen 2022; Calzada Olvera 2022). To make collaboration in such ecosystems possible and successful, there are several requirements that need to be met, including mutual trust, visionary leadership, adequate resources in terms of people and funding, and shared values. These qualities require a sustained commitment across the industry both financially and culturally in order to achieve results (Leonida and Soerensen 2022).

These emerging innovation ecosystems and other forms of multi-stakeholder collaboration are promising steps in overcoming some of the innovation barriers the industry has been struggling with and accelerating innovation and transformation (Byrant 2015; Monitor Deloitte 2016). Some of these barriers include the low appetite for business risk combined with a short-term focus leading to a conservatist “follower” approach to innovation, transactional rather than collaborative relationships with the supply chain, hierarchical, and “siloed” organisational structures with little process integration and command-and-control leadership, and mining companies tending to be consumers of innovation rather than innovate themselves (John Steen et al. 2018; Calzada Olvera 2022).

Each of these barriers could be discussed in more detail; however, there is another barrier to innovation that we consider particularly relevant to new technology development, which is the lack of technology-based start-ups in the mining sector.

### Start-up support

Innovations in the mining sector are mainly driven by mine operators and well-established renowned OEMs and service providers (Calzada Olvera 2022) while a vivid and dynamic start-up culture cannot be observed in the mining sector to date. In 2018, there were some 200 start-ups working on mining projects internationally, with the majority of them based in Australia and the USA (Mining International 2021).

The reasons for this are complex. Some of them include inherent difficulties for innovating in and for the mining sector due to the nature of mining operations—locations are predetermined, operations are fast changing, information of the exact conditions in the mine are uncertain and imperfect, and there is a need for extensive infrastructure and large long-term investments that often hampers investments in innovative technology with uncertain returns. In addition, “innovation in the mining industry has been more strongly driven by a problem-solving approach, than by a technological or market opportunity approach” (Calzada Olvera 2022). Given, however, that every mining operation is unique and every mining system needs to be adapted to the specific conditions of a certain mine, it is challenging for a start-up to identify and understand a particular problem, for which a solution shall be developed. Usually, start-ups emerge in larger numbers if and where the problems to solve are clear and tangible for the developers. In mining, however, the problem is often not clearly defined. In addition, in mining, it is less about developing new products, processes, or services, but about developing solutions that can be integrated into existing systems and processes. Given the unique requirements of individual mine sites, it becomes very difficult to develop “one-size-fits-all” solutions and transfer them among mine sites. This means that solutions must often be specifically adapted, requiring domain-specific specialist knowledge and expertise.

Another challenge to fostering a start-up culture that seems to be inherent and less explicitly addressed in most publications is the poor public image of mining and a widespread lack of awareness of the relevance of raw materials for everyday life and societal development. Consequently, the mining sector is generally not perceived as modern, innovative and hip and not a place to be for start-ups who like to find themselves in fancy co-working spaces equipped with gadgets and work-life offerings. This makes it rather difficult to attract students or at least inspire them to create start-ups in this sector and becoming an entrepreneur is rarely the focus and interest of most of the students enrolled in mining engineering.

The question then becomes how can it be managed to increase the innovation base in mining and, in particular, how can it ensured that potential founders receive technical support from an early stage? While innovation accelerators have emerged in different countries, such as “Expande” (Expande 2022) in Chile or “Kite Company Creators” (Kite Company Creator 2022) in Canada, that are targeting mining start-ups specifically, strong technical support especially through a link with university research centres, as well as fostering of start-ups out of universities directly, is not necessarily provided. However, this is a cornerstone of a recent initiative currently implemented at RWTH Aachen University as part of the ExcellenceStartUp Center.NRW, a government-funded program. The initiative aims at building a leading European tech incubator on the premises of the university and to establish entrepreneurial thinking and action as a central part of teaching and learning culture (RWTH Innovation 2022). One novelty of this initiative lies in building theme-specific innovation ecosystems that are intended to support future founders in product development and with in-depth technology and subject-specific expertise. One of these theme-specific incubators is intended for the area of raw materials, which will be co-located with and managed by the Institute for Advanced Mining Technologies (Institute for Advanced Mining Technologies 2022). 

Creating an infrastructure and environment conducive for start-ups leads to another relevant requirement for technology development, which is an infrastructure to support collaborative technology development and application-oriented research, ideally in partnerships with industry. The next section will outline the need for creating new forms of innovation conducive infrastructures.

### Creating an innovation infrastructure

Just as the development of innovation ecosystems and the promotion of start-ups in the mining sector require structural elements and dedicated organisations or programs to facilitate new forms of partnerships and innovation, the targeted development of technological solutions to industry-driven problems can immensely benefit from physical spaces where universities and industry partners, including mining companies and suppliers, can work together to develop, test, and demonstrate new technologies.

Over the past decade, new instruments for fostering such collaborative forms of innovation have been created, which have been called many different names: regulatory sandboxes or innovation test-beds, real-world laboratories, living labs, innovation spaces, or real-life experiments (BMWK 2019). While the exact definitions vary as much as the terminology itself, generally, regulatory sandboxes and innovation test-beds refer to specific frameworks for testing innovation and regulation for a limited time in a limited area, in which innovative technologies and business models can be tried out in real-life environments, making use of regulatory leeway in order to then inform new legislation around the discovery or innovation (BMWK 2019). Real laboratories, living labs, or real-life experiments generally refer to transdisciplinary research and development facilities and constitute new forms of cooperation between science and civil society that focuses on mutual learning in an experimental setting. The laboratories refer to labs in a social context that include various stakeholders, such as universities, municipalities, NGOs, companies, state institutions, and industry associations that join forces to tackle societal challenges, from sustainability and energy management to mobility, housing, and many other questions (Reallabornetzwerk 2022; Wikipedia 2022; Beecroft et al. 2018).

Within the mining industry, there are some examples of innovative underground test mines, some of which are proprietary, such as the Sandvik test mine in Finland used for rapid prototyping (Sandvik Mining and Rock Technology 2022), while others, such as NORCAT in Canada, have been developed into training and testing facilities for start-ups, small- and medium-sized enterprises, and mining companies offering various testing and training environments and programs as a service to the industry (Norcat 2022). In addition, a limited number of universities have an underground mine setting in a formerly active mine as part of their student learning and research environments, such as the TU Bergakademie Freiberg in Germany (TU Bergakademie Freiberg 2022) and the Colorado School of Mines in the USA (Colorado School of Mines 2021).

In a recently launched initiative, two institutes of RWTH Aachen University, namely the Institute for Advanced Mining Technologies and the Institute for Mineral Processing, have entered a partnership with a local sand mining operation, the Nivelsteiner Sandwerke und Sandsteinbrüche GmbH, and established a collaborative research facility on the premises of the sand mining operation in vicinity to the university. The “Reallabor Nivelstein” combines the approach of regulatory sandboxes and real-world laboratories with the idea of a test mine for application-oriented research as well as innovative teaching formats (Graskz et al. 2022).

Knowing that “structure matters” and that in order for new forms of collaborative innovation, which are required for nurturing a new culture of innovation in the mining sector, also require physical spaces for people to come together and develop and test new solutions and ideas; the “Reallabor Nivelstein” consists of a research hall, equipped with test benches, a co-working space, and a meeting room as well as dedicated testing areas in the production area. In this facility, which has been fully equipped and started research and development in early 2022, technologies can be developed collaboratively with industry in a relevant industrial environment, while the facility itself shall also be developed into a demo factory to showcase the possibilities and capabilities of new technologies to other companies, suppliers, and the public. This includes the actual research hall as well as specific areas of the active mining operation, which can be utilised for testing and demonstration. In addition, the facility constitutes an authentic learning environment for students, where theses can be developed, hackathons organised, and workshops or courses conducted.

Some of the thematic areas that are being addressed at the Nivelstein site, which also hosts the largest PV Power Plant in the state of Northrhine-Westphalia, are sensor-based selective extraction of an autonomously acting dredge, demand-driven controlled sand drying fed, integrating renewable energy sources into mining processes, and process integration and process chain optimisation (Graskz et al. 2022).

The “Reallabor Nivelstein” follows an open and collaborative approach and is generally accessible for other university departments as well who can benefit from the interdisciplinary exchange and collaboration as well as the inherent interdisciplinary nature of the experiments conducted at the facility, which are opening up new possibilities for innovative types of cooperation that can create value for all participants.

As such, the “Reallabor Nivelstein” presents a good example for establishing what Schneidewind et. al have called “long-lasting spaces for transformation and reflexive learning” as well as effective test arenas for collaborative innovation and joint technology development (Schneidewind et al. 2018).

Thinking into the future, environments like the “Reallabor Nivelstein” could also be utilised for piloting a mine of the future and for developing agile support mechanisms in order to support and foster research activities and accompany companies on their journey towards autonomisation and electrification.

While the four sections above have considered relevant prerequisites for technology development and innovation that are linked to, but not directly referring to factors within the mining companies, the last two sections will look at core factors directly related to mining companies.

### (Talent-)Management and the future of work

Adapting management systems and culture have been shown to be an important factor in successfully managing technological transformation processes once the technologies have been selected and acquired. In order for technology to be adopted sustainably and unfold its potential in an operation, adapting work processes and a more agile, responsive company culture have been shown to be key for driving technology-driven change (McKinsey & Company 2018).

With respect to technology development, however, there is another factor, which is the necessity to consider the impact of technology from a workplace perspective from the onset. An often neglected part of technology development is the necessity to include a perspective on human factors, “the human side of mining”, and potential implications, negative or positive, that digitalisation and automation may have, in order to shape the kind of impact technology will have on the operation but also on the workers in particular (Lööw et al. 2019; Lynas 2011; Lööw and Johansson 2020; Halim et al. 2021). As Lööw et al. state, “used correctly, digitalisation can create attractive jobs in safe control room environments, which provide space for the employee’s full expertise and creativity […] but, to succeed […] we must also consider the risks, such as privacy issues, increased stress, and work-life boundaries” (Lööw et al. 2019).

In order to attract a young and diverse workforce that is key to realising the mine of the future, it is therefore necessary to develop and include a comprehensive understanding of the impact of digitalisation and automation on workplace design, diversity, skillsets, working environments, safety, and other factors from the onset. This “human side of mining” is currently investigated comprehensively as part of the NEXGEN SIMS project looking at what the future of work might look like in a “utopian” or rather “dystopian” scenario for the digital miner. The two possible scenarios make it very clear that technological transformation comes with a lot of responsibility and requires mining companies to think carefully about their socioeconomic context. For the more utopian, or positive, scenario to unfold, it will be necessary; Lööw et al. suggest that mining companies “embed all changes in a context of great social responsibility” as the mine “is not limited to the mine” (Lööw et al. 2019). This also implies a change in the commitment of the leadership, a redefinition of the role of mining companies within their socioeconomic context, and an acknowledgement of the responsibility for the scenarios that will define the context and conditions for miners in the future.

### Company culture

The discussion on the future workplaces in mining has already eluded to the fact that the potentially positive effects of new technology are not guaranteed but depend on how they are applied, what the vision and the intention is behind it, and how mining companies position themselves within the larger socioeconomic context and their responsibility towards employees, stakeholders, and society. These are the aspects of company culture that we would like to focus on in the context of technology development and innovation.

On the one hand, this means that work cultures need to be adapted with the aim of creating an environment within the company conducive for innovation and technology adoption by replacing outdated hierarchical structures and siloed thinking with more agile and flat organisational structures. Control and command structures have been firmly established over decades. However, these come with a tendency to focus on the tasks. Especially with regard to attracting the right talent and draw skills from a wider pool, including, for example, more women and people with diverse backgrounds, as well as new data analysts, programmers, and tech talent, there is a pressing need to rethink core aspects of traditional mining management culture (PwC 2017). That is why, in order to prepare for the future, mining companies need to put people first (McKinsey & Company 2020) and create a culture that rewards collaboration and encourages people to work together ((McKinsey & Company 2020; PwC 2017). Leaders for tomorrow must provide vision and purpose and build an inclusive culture of trust and respect (Nel und Treacy 2021). The COVID-19 pandemic has accelerated companies to review work routines, evaluate options for remote work, and possibly outsource certain areas of the operation and thus brought people to the centre of digital transformation. However, mining companies need to embrace this broader and deeper cultural shift in order to attract the right talent and make the most of the technological opportunities that are and will become available.

On the other hand, mining companies need to adopt a different mindset and change their culture with respect to collaboration and partnerships and their position within the larger ecosystem they are embedded in. The complex stakeholder expectations mining companies are facing and which go well beyond the customers, suppliers, and stakeholders they deal with on a daily basis make it an imperative to succeed in building trust with the wider ecosystem of stakeholders, such as investors, regulators, communities, and non-governmental organisations. This can be achieved, for example, by being transparent and practice technology enabled real-time continuous disclosure, by building trust based on listening to stakeholder concerns and acting on them, as well as by successfully applying technology to lessen the environmental and climate footprint, and by sustainably engaging with the communities and create social value beyond corporate boundaries. In addition, collaboration and partnerships with a wider range of partners, as discussed in the section above, can be an avenue to improve public awareness and acceptance of mining (PwC 2017; World Economic Forum 2015; Mitchell 2021). This also means that in order to thrive in the future, a cultural and mindset shift needs to take place that will take mining companies beyond a social license to operate—they will need to start considering themselves as creators of social value and builders of societal capital and position themselves as such. This requires visionary leadership and prioritising people, inside and outside the company (McKinsey & Company 2020; PwC 2017). To close with a quote from BHP CEO Mike Henry, “First and foremost, social value is a way of being and a way of running the business [… it] is embedded in all of the decisions that we make and in the day-to-day way that leaders in BHP lead. There isn’t some unit that handles all social-value activities; this is a line accountability, supported by functional experts. From mine plans to safety decisions to procurement, we’re thinking about how our employees, communities, business partners, and so on are benefitting. Through doing that, we can lift what is already a substantial contribution to our stakeholders further without eroding short-term financial or operational performance. And over time, you build greater value and greater returns for shareholders” (McKinsey & Company 2020).

## Conclusions: towards an expanded notion of Mining 4.0 – human-centred climate smart mining

There are a number of technologies already available that are and will continue to transform the way mining companies operate. Some of the leading digitalisation trends were discussed and reflected on the challenges that partially explain why the full potential of the available technologies has not yet been realised. In addition, specific challenges related to the transition from automating pieces of equipment towards autonomous systems, the current level of implementation, and the prerequisites for advancing towards autonomous systems were discussed. Breakthroughs may likely be expected to become more visible once communication infrastructure and the IIoT get implemented across entire operations, operational processes become digitally integrated across departments and along the value chain, and the full suite of technologies are applied in a targeted and integrated manner. Many challenges still have to be tackled in order to derive substantial value from data.

Given the impressive range of available technologies, it is easy to think that technology is in itself a leveler. However, this paper put forth the argument that while technology is a strong enabler for transformation, there are other non-technological aspects and challenges that constitute a broader context for the current transformation and need to be included in the discussion on the future of mining. Especially when envisioning a mining future that is low carbon with a minimal environmental footprint; digitally integrated and largely autonomous, safe, and socially accepted, technological innovation and breakthroughs in combination with the “human side of mining” are central for this transformation, reflected in the term “Human Centered Climate Smart Mining” to account for an extended notion and concept of future mining.

Making the “human aspects of mining” more explicit adds purpose beyond economic parameters and accounts for the embedded nature of transformational change. Change happens within a context and this context needs to be accounted for more explicitly in the current discussion around creating the autonomous green mine of the future.

In addition, this expanded notion of future mining opens up an opportunity to connect sustainability and technological transformation in an enhanced way. To date, discussions on sustainability in mining are still largely decoupled from discussions on digitalisation (Clausen et al. 2020b). However, technology can be a strong enabler for sustainability and these interrelations can be made more explicit using the concept of the “human centered climate smart mine”.

To conclude, there are breakthroughs required on a technological level, but also on a social, cultural, and societal level in order for mining companies to reposition themselves as builders of social value and societal capital. This, in turn, will be essential for mining companies to remain competitive in the long term. In order to successfully develop and implement the technologies for the autonomous green mine, the human side of mining needs to become central. This can, in turn, contribute to increasing the much-needed social acceptance and attractiveness of the industry, which positively impacts the implementation of technologies and creates a virtuous cycle for change. These kinds of mutually reinforcing effects can lead to breakthroughs in accelerating the transformation much more than technology alone would be able to. 

## Footnotes

1 The keywords “sustainable mining” provide 2254 matches in the OneMine.org Database, “social license” result in 44 matches, and “license to operate” in 229 matches, whereas “emission reduction” results in 330 matches.
